# Natural Course of Clinically Isolated Syndrome: A Longitudinal Analysis Using a Markov Model

**DOI:** 10.1038/s41598-018-29206-y

**Published:** 2018-07-18

**Authors:** Yuli Hou, Yujuan Jia, Jingtian Hou

**Affiliations:** 1 Department of Neurology, First Hospital of Shanxi Medical University, Taiyuan, Shanxi China; 2 Department of Endocrinology, First Hospital of Shanxi Medical University, Taiyuan, Shanxi China

## Abstract

Clinically isolated syndrome (CIS) refers to the initial clinical episode with symptoms suggestive of multiple sclerosis (MS). Due to limited number of long-term follow-up studies, progression pattern from CIS to more advanced stages remains unclear. In the current study, we constructed a Markov model to simulate the natural course of CIS. The model estimated the probabilities of transition from CIS to more advanced disease stages and the duration needed for the progression. The analysis showed: (1) CIS is a solid disease identity: more than 85% of the subjects with a diagnosis of CIS progress to RRMS or more advanced stages within 20 years; (2) the reduction of life expectancy in subjects with CIS is marginal.

## Introduction

Multiple sclerosis (MS) is the most common chronic degenerative disease of the central nervous system (CNS) in young adults^1^. It is caused by axonal demyelination, and characterized by inflammation of the CNS. In nearly 85% of the patients, the disease starts as a single episode referred to as clinically isolated syndrome (CIS), typically affecting the optic nerves, brainstem or spinal cord, during early adulthood^2^. Approximately 84% of the subjects with CIS experience a second clinical demyelinating event and are diagnosed with clinically definite MS (CDMS) within 20 years^3^. With regard to the natural history of CIS, comprehensive cohort studies have revealed that patients diagnosed with CIS may experience four stages: CIS, relapsing-remitting MS (RRMS), secondary progressive MS (SPMS) and death^3–5^. Due to the fact that patients with CIS usually recover from the presenting episode and the uncertainty of long-term prognosis, disease-modifying treatments for CIS remain controversial^6^.

Knowledge of the natural course of CIS can be used to predict disease development as well as to guide the initiation and cessation of disease-modifying treatments. Longitudinal studies of the patient population not receiving treatments are valuable in determining whether a specific disease-modifying treatment is effective. For policy planners, such knowledge can be used to estimate the socioeconomic impact of CIS, to make projections of disability outcomes in the population, and to implement appropriate health care management^1,7^.

Existing studies of the natural course of CIS are sparse and limited by small sample size. In the current study, we used a Markov model to synthesize available information about the natural course of MS after CIS. Markov model is a probability model based on the development of a transition matrix that describes the probabilities of change from one stage to another^8^. If a disease is adequately stratified into distinct stages, a Markov model could be used to predict the transition (either progression or regression) from one stage to another^9–11^. Markov model has been used to study a variety of diseases, including cancer, diabetic retinopathy, HIV infection and sepsis^9,12–14^. However, majority of the available long-term follow-up MS studies used survival analyses to assess the natural course^5,15,16^. In this study, we synthesized data from long-term population-based cohort studies using consensus recommendations. A Markov model was used to simulate the natural course of MS from the initial episode of CIS, and to predict the transition probabilities, life expectancies and theoretical prognoses of patients who had not been treated prophylactically with any disease-modifying drugs.

## Methods

### Disease states

With increasing number of comprehensive cohort studies on the natural course of MS, the international classification of MS (CIS, RRMS, SPMS, primary progressive MS, and progressive-relapsing MS) has become widely accepted^17^. CIS is defined as the first clinical episode in which a patient has symptoms and signs suggestive of MS. CIS is isolated in time and space. RRMS is characterized by “clearly defined relapses with full recovery or with sequelae and residual deficit upon recovery; periods between disease relapses characterized by a lack of disease progression.” SPMS is defined as an “initial RR disease course followed by progression with or without occasional relapses, minor remissions, and plateaus.” Death is an absorbing state from which transition to other states cannot occur.

**Figure 1:**
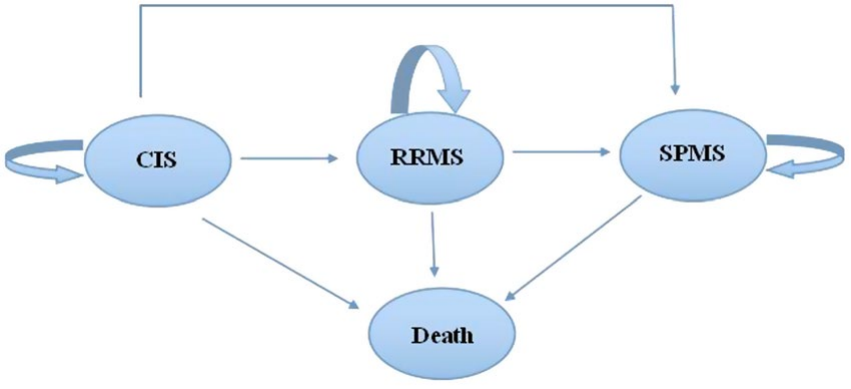
The possible transitions from CIS during disease progression. CIS: clinically isolated syndrome; RRMS: relapsing-remitting multiple sclerosis; SPMS: secondary progressive multiple sclerosis.

Patients diagnosed with CIS may eventually experience RRMS, SPMS and death^5^. A given subject could transition from one state to another (Fig. 1). For example, over a given time interval, a patient with CIS may remain in the CIS state, progress to the RRMS or SPMS state, or die. Similarly, a patient diagnosed with RRMS may remain in the RRMS state, progress to SPMS, or die over a time period, whereas a patient with SPMS may remain in the SPMS state or die.

### Statistical analysis

We constructed a Markov model that included three widely accepted states (CIS, RRMS, and SPMS), as well as the absorbing state (death)^18^. The Markov model was used to estimate the life expectancy of patients and the duration required for disease progression from one to another state^8,11^. Briefly, the model uses a stochastic processing method in which transitions and transition times from the current state are assumed to be conditionally independent of the previously occupied state^19^, based on the previously described principle^9^. For example, once a patient in the CIS state progresses to RRMS, the subsequent transition depends only on the current state (RRMS) and not the former state (CIS). To construct the Markov model of CIS, we focused on long-term population-based cohorts of patients with CIS, RRMS and SPMS.

We systematically reviewed published literature and obtained CIS transition probabilities from pre-existing natural history cohorts. Majority of the studies reported transition data over 10-year intervals. Thus, we used 10-year interval data for longitudinal analysis of CIS using the Markov model, specifying the unit time interval as 10 years. The probability of remaining at the same stage and that of transitioning to a different stage within 10 years were obtained from the published studies. The probability of transitioning from one state to another was calculated from the observations according to Beck^8,20^ using the formula *Rij* = *Nij*/*Ni*, where *Nij* is the number of patients who transitioned from stage i to stage j during a specific interval, and *Ni* is the total number of patients who started in stage i. Table 1 summarizes the characteristics of the included studies. Table 2 provides the 10-year transition data and probabilities obtained from these published studies.

**Table 1:** Characteristics of the longitudinal follow-up studies included in the analysis.

Author	Year	Diagnostic criteria	Initial status	Number of patients (n)	Female/Male	Age of onset	Follow-up (years)
I. O’ Rordan^3^	1998	Definite	CIS	81	53/28	32.3	10
L. K. Fisniku^4^	2008	CIS or possible		110	73/37	32.0	20
M. Eriksson^15^	2003	MS Poser		308	186/122	NA	25
P. A. Brex^21^	2002	criteria		71	49/22	32.0	14
S. J. Pettock^34^	2004	Poser	RRMS	48	NA	NA	10
H. Tremlett^35^	2009	criteria		2454	1457/997	NA	25
H. Tedeholm^5^	2015			212	137/75	NA	50
J. Kuhle^30^	2015	Poser	SPMS	176	NA	41	10
N. G. Torkildsen^31^	2008	criteria		878	545/333	32.9	50

Initial status: the status of each patient cohort at the onset of longitudinal follow-up. CIS: clinically isolated syndrome; RRMS: relapsing-remitting multiple sclerosis; SPMS: secondary progressive multiple sclerosis. NA: not available.

**Table 2:** Transition data and probabilities at 10-year interval.

Author	Initial status	N	n (%) after 10-years follow-up			
			CIS	RRMS	SPMS	Death
J. I. O’ Riordan^3^	CIS	81	29 (35.8)	39 (48.2)	12 (14.8)	1 (1.2)
H. Tedeholm^5^	RRMS	212	—	121 (57.1)	87 (41.0)	4 (1.9)
J. Kuhle^30^	SPMS	176	—	—	163 (92.6)	13 (7.4)

Initial status: the status of each patient cohort at the onset of longitudinal follow-up. n: number of patients;

CIS: clinically isolated syndrome; RRMS: relapsing-remitting multiple sclerosis; SPMS: secondary progressive multiple sclerosis.

Diagnostic criteria: The diagnostic criteria of each natural history study are shown in Table 1. Briefly, the CIS cohorts were diagnosed as definite CIS or possible MS, whereas the Poser criteria do not include the diagnostic criteria of CIS. Among the 4 long-term follow-up studies of CIS, the CIS cohort was diagnosed according to “possible MS” in the study of Maja Eriksson^15^, meaning “patients who had characteristic symptoms such as unifocal optic neuritis, typical reversible sensory disturbance or internuclear ophthalmoplegia, without evidence of dissemination in space and time”. In the remaining 3 studies^3,4,21^, “definite CIS”, defined as “the first clinical episode in which a patient has symptoms and signs suggestive of MS, always isolated in time and also clinically isolated in space” was used. Definitions of RRMS and SPMS were based on the Poser criteria and consistent across all studies included in the analysis^22^.

**Figure 2:**
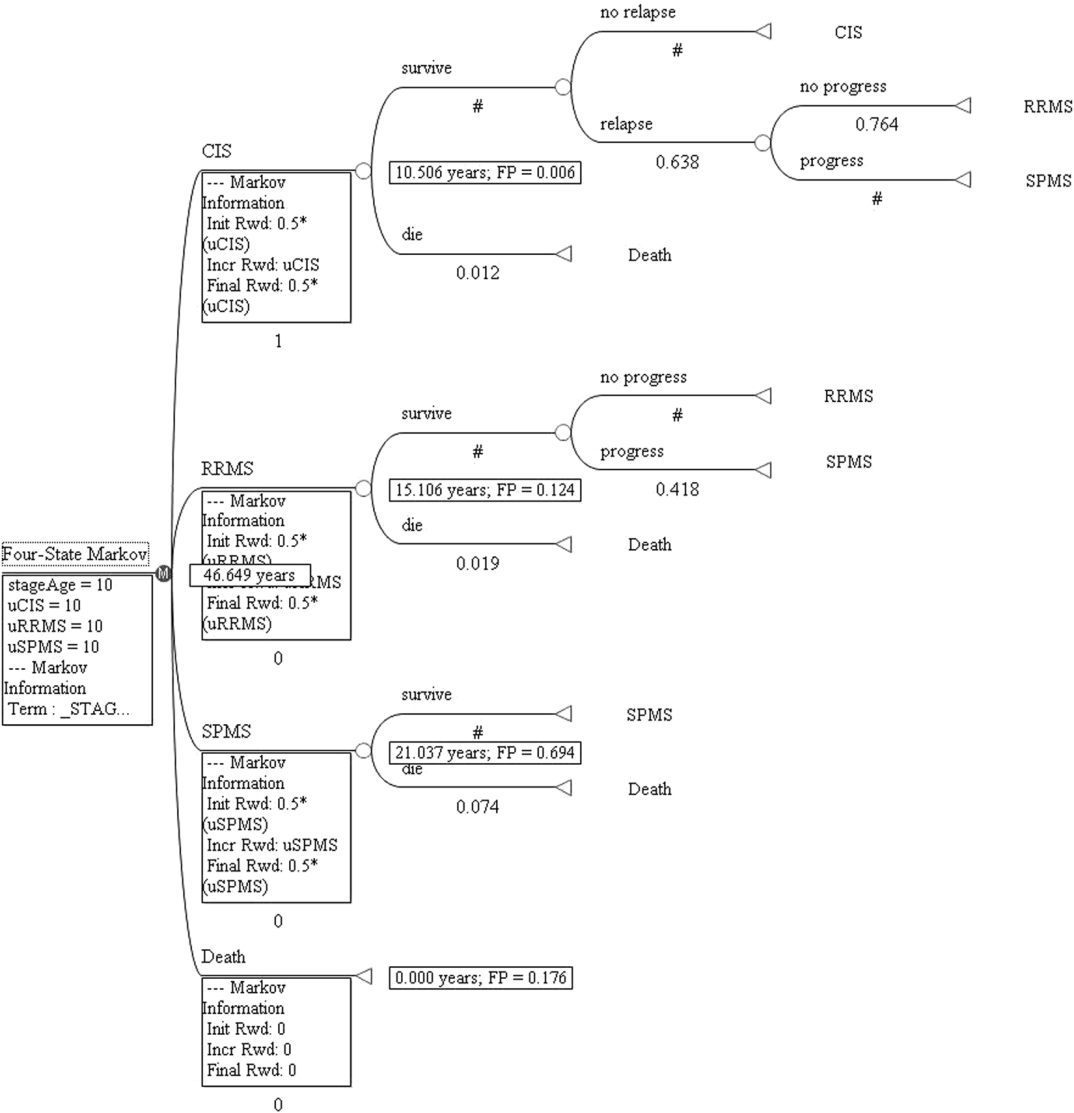
Markov tree of the transitions among the four disease states. A Markov tree was constructed using TreeAge Pro 2011 software. Since the disease involves four stages, the Markov model was referred to as a “Four-state Markov” model (left-most box). CIS: clinically isolated syndrome; RRMS: relapsing-remitting multiple sclerosis; SPMS: secondary progressive multiple sclerosis; FP: final probability; uCIS, uRRMS, and uSPMS: health utility values; Init Rwd: initial health utility value; Incr Rwd: increasing health utility value; Final Rwd: final health utility value. #: rest (no transition) probability; 0.5*: half-cycle correction.

We used TreeAge Pro 2011 software to construct a Markov tree (Fig. 2). The 10-year transition probabilities are shown in Table 2. We referred to the health utility values of the CIS, RRMS and SPMS states as uCIS, uRRMS and uSPMS, respectively. For estimation of average life expectancy, the health utility value was set at 10 for each survival state, considering the 10-year model cycle. In an absorbable Markov process, life expectancy is typically overestimated by a half cycle due to unadjusted expectations in both the starting cycle and subsequent cycles. To address this issue, we used half-cycle correction to correct for overestimation. The average life expectancy in most countries has been reported at 70–80 years^23^. CIS onset typically occurs at 20–30 years of age^18^. Accordingly, we performed calculations with cycle periods of 10, 20, 30, 40 and 50 years and obtained transition probabilities and average life expectancy at 10, 20, 30, 40 and 50 years after CIS onset.

## Results

### Duration of each state and life expectancy

A Markov tree was constructed based on the possible progression of the disease (Fig. 2). The results revealed that over a 50-year period, patients initially diagnosed with CIS typically progressed to RRMS over a 10-year interval. The estimated duration of RRMS was approximately 15 years. The estimated duration of SPMS was approximately 21 years. The estimated life expectancy of the CIS patients was 46.6 years over a period of 50 years after CIS onset.

### Transition probabilities

The estimated transition probabilities between the states over a 10-year interval and average life expectancy are reported in Table 3. We estimated the probability of remaining in the initial state, namely CIS, as well as the probability of transitioning to another state over successive decades. At 10 years after the CIS onset, an estimated 35.8% (95%CI: 25.1–46.5%) remained in CIS, 48.1% (95%CI: 37.0–59.3%) transitioned to RRMS, and 14.9% (95%CI: 6.9–22.7%) progressed to SPMS. Consistent with published data^18^, 1.2% (95%CI: 0.0–7.1%) of the initial CIS individuals died within the first 10 years. The estimated duration of CIS during the first decade was 9.88 years. At 20 years after CIS onset, 12.8% (95%CI: 5.0–19.7%) remained in CIS, 44.7% (95%CI: 33.4–55.5%) developed RRMS, 38.8% (95%CI: 27.5–49.1%) developed SPMS, and 3.6% (95%CI: 0.8–11.1%) died. The estimated duration of the second stage was 9.63 years, and the cumulative life expectancy at this stage was 24.51 years. These estimated probabilities are consistent with that reported by Fisniku^4^, who reported progression into CDMS in 81% of the subjects within 20 years. At 50 years after CIS onset, an estimated 69.4% (95%CI: 58.9–79.4%) of the patients were in the SPMS state; an estimated 12.4% (95%CI: 5.0–19.7%) of the patients transitioned to RRMS, whereas 17.6% (95%CI: 8.9–25.7%) died of severe CIS complications. The estimated duration of this stage was 4.12 years, and the cumulative life expectancy was 46.64 years. The estimated mortality in the current study was consistent with that reported by Torkildsen^18^, who estimated 22.5% mortality at 50 years.

**Table 3:** Transition probabilities and life expectancy of patients with CIS.

Stage	State	Probability(95%CI)	Stage Reward	Cumulative Reward
Stage 0			5	5
	CIS	1	0	
	RRMS	0	0	
	SPMS	0	0	
	Death	0	0	
Stage 1			9.88	14.88
	CIS	0.358 (0.251, 0.465)	3.58	
	RRMS	0.481 (0.370, 0.593)	4.81	
	SPMS	0.149 (0.069, 0.227)	1.49	
	Death	0.012 (0.000, 0.071)	0	
Stage 2			9.63	24.51
	CIS	0.128 (0.050, 0.197)	1.28	
	RRMS	0.447 (0.334, 0.555)	4.47	
	SPMS	0.388 (0.275, 0.491)	3.88	
	Death	0.036 (0.008, 0.111)	0	
Stage 3			9.25	33.76
	CIS	0.046 (0.014, 0.128)	0.46	
	RRMS	0.317 (0.206, 0.411)	3.17	
	SPMS	0.562 (0.458, 0.678)	5.62	
	Death	0.075 (0.016, 0.132)	0	
Stage 4			8.76	42.52
	CIS	0.016 (0.000, 0.054)	0.16	
	RRMS	0.203 (0.109, 0.286)	2.03	
	SPMS	0.657 (0.549, 0.760)	6.57	
	Death	0.123 (0.050, 0.197)	0	
Stage 5			4.12	46.64
	CIS	0.005 (0.000, 0.047)	0.03	
	RRMS	0.124 (0.050, 0.197)	0.62	
	SPMS	0.694 (0.589, 0.794)	3.47	
	Death	0.176 (0.089, 0.257)	0	

CIS: clinically isolated syndrome; RRMS: relapsing-remitting multiple sclerosis; SPMS: secondary progressive multiple sclerosis. Stage: cycle period, each cycle period refers to 10 years; e.g., “Stage 0” refers to the period before the first cycle, and “Stage 1” refers to the first cycle. State: disease state. Probability: transition probability at each stage. Stage reward: life expectancy at each stage. Cumulative reward: cumulative life expectancy at each stage.

**Figure 3:**
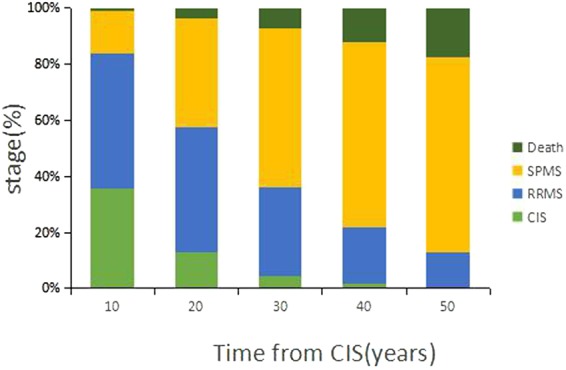
Percentage of the subjects at each of the disease stages at varying time after CIS onset. CIS: clinically isolated syndrome; RRMS: relapsing-remitting multiple sclerosis; SPMS: secondary progressive multiple sclerosis.

Figure 3 illustrates the percentage of the subjects at each of the disease stages at varying time after CIS. At 10 years after CIS onset, the percentage of the subjects at each of the 4 disease stages (CIS, RRMS, SPMS, death) was 35.8% (95%CI: 25.1–46.5%), 48.1% (95%CI: 37.0–59.3%), 14.9% (95%CI: 6.9–22.7%), and 1.2% (95%CI: 0.0–7.1%), respectively. At 20 years, the percentage at each of the 4 disease stages was 12.8% (95%CI: 5.0–19.7%), 44.7% (95%CI: 33.4–55.5%), 38.8% (95%CI: 27.5–49.1%), and 3.6% (95%CI: 0.8–11.1%), respectively. At 50 years after CIS onset, only 0.5% (95%CI: 0.0–4.7%) of patients remained in the CIS state, whereas an estimated 69.4% (95%CI: 58.9–79.4%), 12.4% (95%CI: 5.0–19.7%), and 17.6% (95%CI: 8.9–25.7%) of patients transitioned into the SPMS, RRMS, and death, respectively. The proportion of CIS patients declined over time in an exponential manner. Most of the patients transitioned to RRMS between the first and third decades, and the proportion of CIS patients who transitioned to clinically defined MS declined steadily with the progression of the disease.

## Discussion

With advances in disease-modifying treatments for MS, there has been great interest and research regarding patients with CIS. Much progress has been made in terms of understanding the cause, pathogenesis and risk factors of CIS^24–27^. CIS is a chronic disease with an extended trajectory. Also, patients with CIS typically recover from their presenting episode. Accordingly, thorough understanding of the natural course of CIS is needed to balance the potential benefits and adverse effects of disease-modifying treatments.

The Markov model is particularly suitable for simulating the natural course of CIS. The Markov model could also provide a comprehensive view of the disease process, and facilitate estimation of the proportions of individuals in different states at future time points and the duration of a particular state, thus allowing for efficient use of incomplete information when disease histories were available for only a small proportion of study participants.

CIS episodes are typically mild, and many individuals recover completely without therapeutic intervention^6^. Prediction of the long-term course of CIS from disease onset is challenging. In addition, the use of disease-modifying treatments for CIS is controversial because of the uncertainty concerning the long-term clinical prognosis and the benefits and adverse effects of various treatments, although such treatments have the potential to delay the transition from CIS to definite MS^27–29^. The results of the current study using a Markov model are generally consistent with the findings reported by previous longitudinal studies^3–5,30,31^. By 50 years after CIS onset, an estimated 69.4% (95%CI: 58.9–79.4%) of the patients had converted to SPMS, 12.4% (95%CI: 5.0–19.7%) had transitioned to RRMS, and 17.6% (95%CI: 8.9–25.7%) had died of complications. The cumulative life expectancy was estimated to be 46.6 years. Thus, CIS significantly reduces quality of life but has little impact on overall survival of the patients. European^32^ and North American^33^ placebo-controlled studies of disease-modifying treatments for SPMS have reported conflicting results. A 10-year follow-up study of an European multicenter randomized controlled trial of interferon beta (IFN-β)-1b failed to produce long-term benefits in SPMS patients^30^. Therefore, to prevent future tissue damage and to delay the conversion to CDMS in patients with CIS, the use of disease-modifying treatments should be considered, particularly for agents with favorable safety profile.

There are several limitations in the current study. The estimation is not based on a “true” model: prediction and simulation of the short-term course of CIS are limited due to a lack of original data on short-term transition probabilities. In addition, the paucity of data also prevents evaluation of the effects of potential confounding factors, such as age of onset and gender, on disease progression. Also, treatment is a critical and modifiable factor that could theoretically affect the outcome. Studies that elaborated the treatment, however, are typically short-term. At this point of time, we are not able to assess the treatment effects on disease progression. Thus, better predictors of long-term prognosis are urgently needed to enable early targeting of treatments for patients who are most likely to experience long-term therapeutic benefits. The lack of precise and effective long-term predictors is accompanied by a limited understanding of the mechanisms underlying the variable longitudinal course of CIS.
